# Recovery of Waste Materials: Technological Research and Industrial Scale-Up

**DOI:** 10.3390/ma15020685

**Published:** 2022-01-17

**Authors:** Franco Medici

**Affiliations:** Department of Chemical Engineering, Materials and Environment, Faculty of Civil and Industrial Engineering, “Sapienza” University of Roma, Via Eudossiana 18, 00184 Roma, Italy; franco.medici@uniroma1.it

An increase in population, booming economy, rapid urbanization and the rise in living standards have exponentially accelerated waste production. In this scenario, solid waste management is one of the fundamental problems of our society and constitutes a symbol of its limits and inefficiency.

Currently, 2 billion tons per year of municipal solid waste are produced worldwide and, at least, about 33% of this amount remains uncollected by the different municipalities. Recent research estimated that the production of this waste could reach 3.4 billion tons per year in 2050. However, waste production as a whole concerns different streams and origins other than municipal solid waste, including industrial, agricultural, construction and demolition, hazardous, medical and electronic waste. Production estimates of these latter types of waste are more uncertain because the source of production is differentiated and widespread. However, industrial waste generation is almost 18 times greater than municipal solid waste, and global agricultural waste production is more than 4 times the municipal solid waste. Several solutions have been proposed regarding the problem, for example, the theoretical approach of “zero waste”, which is a philosophy that encourages the redesign of resources’ life cycles so that all products can be recycled. In a zero-waste system, material flow is circular, and the same materials are used over and over again until the optimum level of consumption is reached. Moreover, the practice of circular economy includes our lifestyles, social organization and the restructuring of industrial production.

In light of this, the main problem that we have posed in the Special Issue “Recovery of waste materials: technological research and industrial scale-up” is to offer a minimum contribution to the solution of a huge problem, by presenting some findings for the recovery and the recycling of industrial waste. Contributions, coming from three continents and several countries (Canada, Germany, Korea, India, Italy, Poland, Saudi Arabia, Spain and Sweden), were collected from the research community illustrating the current direction and innovative advances in the field of waste materials.

Published papers as a whole concern different waste materials such as the recovery of different building materials [1,2,3,4], the treatment of waste deriving from electrical and electronic equipment [5,6,7], the utilization of stainless-steel slags [8,9], agricultural and domestic waste [10,11] and plastics [12].

The largest group of contributions concerns the recovery of waste materials in the construction sector, one paper (Gomez Escobar V. et al.) [1] considers the reuse of cigarette butts as acoustic absorbers in building constructions, through a preliminary process of chemical cleaning to eliminate the major metal ions as well as organic pollutants present in the samples. After the cleaning procedure, samples present higher adsorption coefficients than those of non-cleaned samples, this is due to an increase in the adsorption surface of the filters’ fibers after the cleaning procedure.

Abis M. et al. [2] highlight that the main obstacle in reusing the bottom ash from municipal solid waste incinerators as a recycling aggregate to be used as a construction material is the content of salts and potential toxic elements concentrated in a layer that coats the bottom ash particles. In this work, a dry treatment process based on abrasion for the removal of salts and other elements is presented. A third paper (Malek M. et al.) [3] shows that the steel chips generated by lathes and mill machines are difficult to recycle, but this steel waste can be conveniently reutilized as a replacement for fine aggregate to produce concrete. Lastly, Martinez-Garzia R. et al., in a review paper, [4] present an overview of the bibliographic status of the design parameter’s influence on the mix proportion of self-compacting concrete with recycled aggregate derived from construction and demolition waste.

Waste deriving from Electrical and Electronic Equipment (WEEE) is growing significantly all over the world; regarding this, Ippolito N.M. et al. [5] considered the recovery of gold from the printed circuit boards of used mobile phones, as well as silver and palladium, making them among the most valuable components of WEEE. Their recycling would also reduce the environmental impact of this waste due to the presence of heavy metals like copper and nickel. Two different hydrometallurgical routes for the recovery of copper and then gold were tested, moreover a flow sheet of the process was proposed. In another study, Santucci V. and Fiore S. proposed two papers [6,7], the first one considered the valorization of polyurethane foam (PUF) deriving from end-of-life refrigerators through a simple sieving process to obtain three fractions to be used as oil adsorbent. Particularly, the obtained fine fraction (d < 0.71 mm) revealed oil sorption performance at least 3–4 times higher than that of commercial products. Furthermore, the second one explored the performance of such waste (PUF) as an adsorbent for wastewater treatment after a pre-treatment sieving and washing, concluding that this waste could be applied “rough cut”.

Jarnerud T. et al. [8], more or less in the same topic, have studied how CaO-containing waste from pulp and paper industries such as fly ash and calcined lime mud can be utilized to neutralize and purify wastewaters from the pickling processes in steel mills. De Colle M. et al. [9] proposed the utilization of stainless steel slags for the pH buffering of acidic wastewaters.

Recycling biomass and different types of organic waste constitutes a way of increasing the share of renewable sources in energy production; in fact, the Sustainable Development Goals set out by the United Nations highlight renewable energy as a key to the success of Agenda 2030. In this framework Kosakowsky W. et al. [10] studied the thermo-chemical decomposition of six types of agricultural waste biomass. The biomass conversion process was studied under a condition of limited oxygen in the reactor, and the temperature was raised from 450 to 850 °C for over 30 min, followed by a residence time of 60 min. Moreover, the obtained biochars present a combustion heat and a calorific value much higher compared to the biomass from which they were made and comparable to a good quality coal.

The last two papers are review works. Yadav K. V. et al. [11] consider agricultural, industrial and household waste that have the potential to generate value-added products, more specifically, industrial waste as fly-ash, gypsum waste and red mud can be used for the recovery of alumina, silica and zeolite. Agricultural waste materials, which are mainly organic, are biodegradable and can be used for the development of carbon-based materials and activated carbon. Furthermore, domestic waste, such as incense stick ash and eggshells, which is rich in calcium can be used as a potential source of either calcium oxide or carbonate.

From a sustainability point of view, the conversion of plastic waste to fuel or, better yet, to individual monomers, leads to a much greener waste management compared to landfill. Following this approach Papari S. et al. [12] reviewed the potential of pyrolysis as an effective thermo-chemical conversion method for the valorization of plastic waste. This waste, which can be a source of detrimental problems to terrestrial and marine ecosystems, can be thermochemically converted into valuable products, such as gasoline, diesel and wax.

In conclusion, the published works demonstrate a scientific and technological relevance to the topics dealt with, but the problems addressed in this Special Issue go beyond any solution that the scientific community is able to propose.

In fact, the “Industrial system, at the end of its cycle of production and consumption, has not developed the capacity to absorb and reuse waste and by-products. We have not yet managed to adopt a circular model of production capable of preserving resources for present and future generations, while limiting as much as possible the use of non-renewable resources for present and future generations, moderating their consumption, maximizing their efficient use, reusing and recycling them. A serious consideration of this issue would be one way of counteracting the throwaway culture which affects the entire planet, but it must be said that only limited progress has been made in this regard”. (Encyclical Letter, Laudato Sì, Holy Father Francis-Bergoglio J.M., 2015).

## Data Availability

Not applicable.
